# The complete mitochondrial genome of jujube leaf roller, *Ancylis sativa* (Lepidoptera: Tortricidae) with phylogenetic implications

**DOI:** 10.1080/23802359.2025.2576516

**Published:** 2025-10-28

**Authors:** Dongping Cao, Bo Hong, Li Dai, Yingyan Zhai, Tianqi Tian, Feng Zhang

**Affiliations:** ^a^ Forestry Workstation of Yulin City, Yulin, China; ^b^ Bio-Agriculture Institute of Shaanxi, Shaanxi Academy of Sciences, Xi’an, China; ^c^ Forestry & Grassland Pest Control and Fire Prevention Center of Dingbian County, Yulin, China

**Keywords:** *Ancylis sativa*, jujube, gene arrangement, bootstrap value

## Abstract

In this study, the complete mitochondrial genome of *Ancylis sativa* was sequenced and assembled. The circular genome is 15,262 bp in length and comprises 37 genes: 13 protein-coding genes (PCGs), 22 transfer RNA (tRNA) genes, and two ribosomal RNA (rRNA) genes, along with one AT-rich region. The average nucleotide composition is A = 40.95%, T = 38.04%, C = 13.01%, and G = 8.00%, resulting in an A + T content of 78.99%. Phylogenetic analysis indicated that *Ancylis sativa* was closely related to *Ancylis unculana* in the subfamily Olethreutinae, which can enhance the evolutionary and phylogeographic study of *Ancylis sativa*.

## Introduction

**Figure 1.**
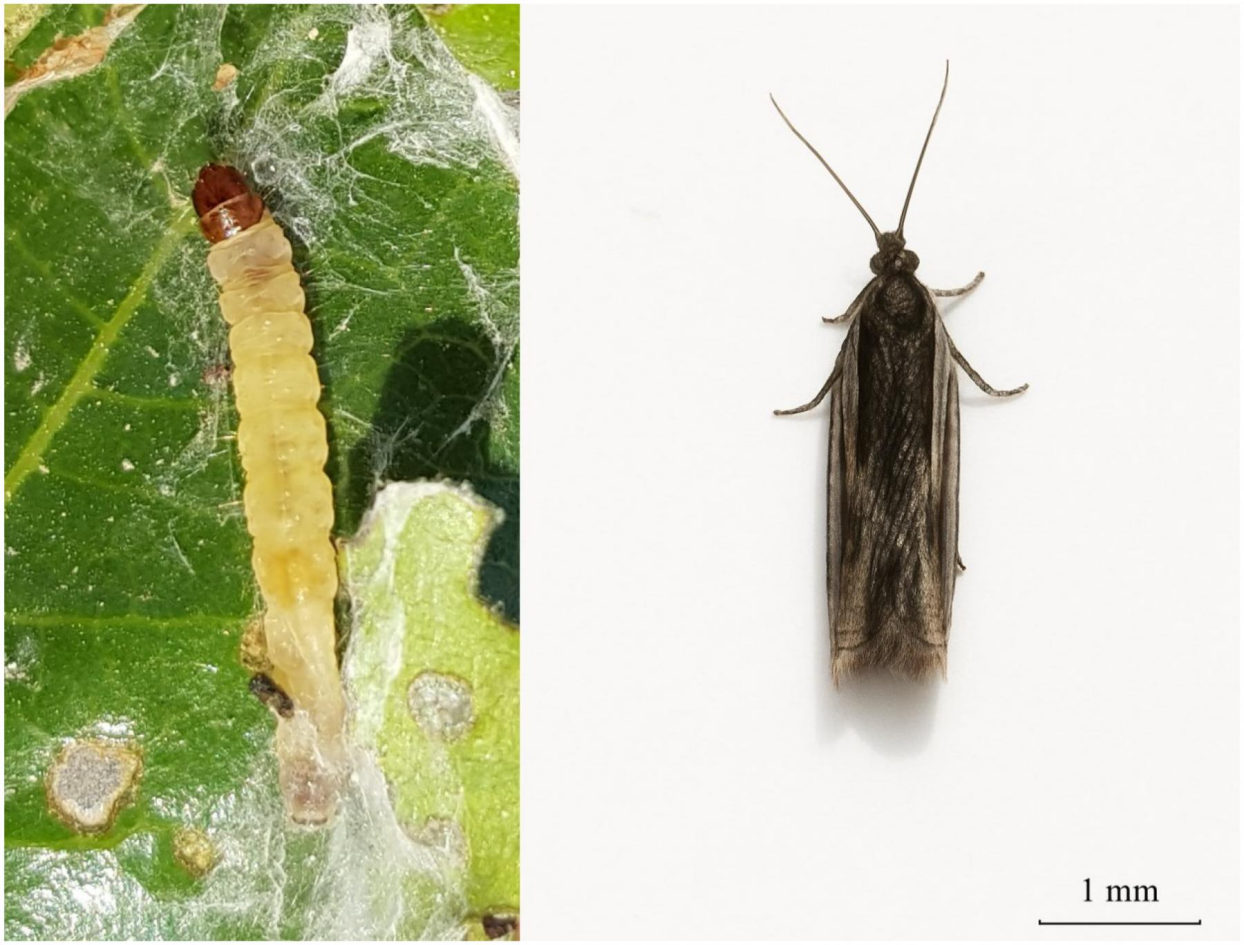
Larva and adult images of *Ancylis sativa* by Dongping Cao.

The jujube leaf roller, *Ancylis sativa* Liu 1979 (Lepidoptera: Tortricidae) is a major pest that feeds only on jujube leaves. It is mainly distributed in China (including Hebei, Henan, Shanxi, Shandong, Shaanxi, Jiangsu, Zhejiang, Anhui, Hubei, and Hunan provinces) (Liu 1979; Li 1992), North Korea, South Korea, Japan, India (Byun 2011), and Pakistan (Nizamani et al. 2016). Adults of *Ancylis sativa* possess 6–7 mm-long yellowish-brown bodies and have a wingspan of approximately 14 mm. While the anterior half of the forewing appears light tan, the posterior region manifests as black with glossy, dark-brown scaled texture. The distal margin terminates in a pronounced hook. Larvae measure 13–15 mm in length with light brown heads (Figure 1). During spring leaf emergence in jujube trees, larvae infest young buds and emerging foliage, binding leaves with silk to form feeding shelters (Li 1992; Luo 2015). This behavior has caused considerable economic losses to the jujube industry. In recent years, multiple investigations have addressed the biology, population dynamics, and management approaches of *Ancylis sativa* (Chen et al. 2016; Nizamani et al. 2016; Zhang et al. 2022). Nevertheless, a complete mitochondrial genome sequence for *Ancylis sativa* remains absent from the NCBI database. In this study, we characterized the complete mitogenome of *Ancylis sativa* through sequencing, assembly and annotation, followed by comparative phylogenetic analyses of Tortricidae species to reveal evolutionary patterns.

## Materials and methods

### Sample collection and genomic DNA extraction

The specimen of *Ancylis sativa* was collected from jujube trees in Qingjian County, Yulin City, Shaanxi Province, China (110.4690° E, 37.0759° N, altitude 754 m). The specimen was deposited in the Laboratory of Pest Monitoring and Control Center, Bio-Agriculture Institute of Shaanxi, Xi’an, Shaanxi, China (http://swny.ac.cn/, contact person and email: Feng Zhang, zhangfeng73@xab.ac.cn) under the voucher number SN2023ZNC01. Genomic DNA was isolated from the thoracic muscle tissue of male adult using the TIANamp Genomic DNA Kit (Tiangen, Beijing, China) and stored at −20 °C.

### Mitochondrial genome sequencing, assembly, and annotation

The sequencing library was sequenced on an Illumina NovaSeq X Plus platform using paired-end technology (150 bp × 2). The mitogenome sequence was assembled using MITObim v1.9.1 (Hahn et al. 2013) with the species *Ancylis unculana* (GenBank accession no. MT950533) (Yang et al. 2022) as the reference genome. The alignment BAM file was generated by Bowtie2 v2.3.4 (Langmead and Salzberg 2012) using the clean paired-end FASTQ files and the reference genome, and sequencing depth and coverage information were calculated using SAMtools v1.7 (https://github.com/samtools/samtools; Danecek et al. 2021). Approximate gene boundaries were delineated by the MITOS2 Web Server (http://mitos2.bioinf.uni-leipzig.de/index.py; Bernt et al. 2013). Nucleotide sequences and positions of 13 protein-coding genes (PCGs), 22 transfer RNAs (tRNAs), and two ribosomal RNAs (rRNAs) were subsequently validated using Geneious Prime 2021.1 (Kearse et al. 2012). The circular genome map was visualized with Proksee (https://proksee.ca/; Grant et al. 2023), and nucleotide skew values were calculated using the formulas: AT-skew = (A − T)/(A + T), GC-skew = (G − C)/(G + C) (Perna and Kocher 1995).

### Phylogenetic analysis

Mitogenome sequences of 38 species, including 36 species in the family Tortricidae (including *Ancylis sativa*) and two outgroup species *Bombyx mori* Linnaeus, 1758 and *Mythimna separata* Walker, 1865, were downloaded from the GenBank database (Table 1). All 13 PCG nucleotide sequences were aligned with MAFFT v7.037 (L-INS-i strategy; Katoh et al. 2002), concatenated using PhyloSuite v1.2.3 (Zhang et al. 2020), and subsequently employed for phylogenetic tree construction. Using the GTR + I + G model (selected as best-fit), we generated a maximum-likelihood phylogeny via IQ-TREE v2.2.0 (Nguyen et al. 2015) with 10,000 ultrafast bootstrap replicates. Final tree visualization was rendered using FigTree v1.4.4.

**Table 1.** Accession number and reference information for 38 species.

GenBank accession no.	Species	Subfamily	Length (bp)	References
MW924665	*Acleris fimbriana*	Tortricinae	15,558	Yang et al. (2021)
MW413307	*Cochylidia moguntiana*	Tortricinae	15,433	Zhao et al. (2021)
MW413306	*Cochylimorpha cultana*	Tortricinae	15,348	Qi et al. (2021)
MW491464	*Eugnosta dives*	Tortricinae	15,392	Unpublished
DQ073916	*Adoxophyes honmai*	Tortricinae	15,680	Lee et al. (2006)
JX872403	*Adoxophyes orana*	Tortricinae	15,343	Wu et al. (2013)
MW924658	*Archips betulanus*	Tortricinae	16,089	Yang et al. (2021)
MW924656	*Archips podanus*	Tortricinae	15,461	Yang et al. (2021)
MG944241	*Choristoneura conflictana*	Tortricinae	15,541	Fagua et al. (2018)
OP747297	*Choristoneura metasequoiacola*	Tortricinae	15,128	Liang et al. (2023)
MG948543	*Choristoneura murinana*	Tortricinae	15,540	Fagua et al. (2018)
MG932647	*Choristoneura fumiferana*	Tortricinae	15,541	Fagua et al. (2018)
MG948540	*Choristoneura biennis*	Tortricinae	15,533	Fagua et al. (2018)
MG948539	*Choristoneura occidentalis*	Tortricinae	15,536	Fagua et al. (2018)
MG944242	*Choristoneura pinus pinus*	Tortricinae	15,536	Fagua et al. (2018)
MG948544	*Choristoneura rosaceana*	Tortricinae	15,544	Fagua et al. (2018)
HQ452340	*Choristoneura longicellana*	Tortricinae	15,759	Wu et al. (2016)
MZ636560	*Clepsis pallidana*	Tortricinae	15,679	Song et al. (2022)
KJ508051	*Epiphyas postvittana*	Tortricinae	15,451	Timmermans et al. (2014)
MT499230	*Cerace xanthocosma*	Tortricinae	15,344	Ding et al. (2020)
OQ865260	*Ancylis sativa*	Olethreutinae	15,262	This study
MT950533	*Ancylis unculana*	Olethreutinae	15,522	Yang et al. (2022)
MW924662	*Bactra venosana*	Olethreutinae	15,588	Yang et al. (2021)
MT548574	*Celypha flavipalpana*	Olethreutinae	15,691	Xiang (2020)
MK962620	*Phiaris dolosana*	Olethreutinae	15,562	Li et al. (2019)
KP677508	*Lobesia botrana*	Olethreutinae	15,229	Piper et al. (2016)
MK820027	*Eudemis lucina*	Olethreutinae	16,056	Zhuang et al. (2019)
JX407107	*Cydia pomonella*	Olethreutinae	15,253	Shi et al. (2013)
MH013480	*Leguminivora glycinivorella*	Olethreutinae	15,506	Unpublished
MT165691	*Grapholita delineana*	Olethreutinae	15,599	Song et al. (2021)
KJ671625	*Grapholita dimorpha*	Olethreutinae	15,813	Niu et al. (2016)
HQ392511	*Grapholita molesta*	Olethreutinae	15,717	Son and Kim (2011)
MW755978	*Thaumatotibia leucotreta*	Olethreutinae	15,394	Jukes (2021)
KF498969	*Retinia pseudotsugaicola*	Olethreutinae	15,282	Unpublished
JX028270	*Rhyacionia leptotubula*	Olethreutinae	15,877	Zhu et al. (2012)
MH013482	*Loboschiza koenigiana*	Olethreutinae	15,440	Unpublished
AB070264	*Bombyx mori*	Bombycinae	15,656	Yukuhiro et al. (2002)
KF730242	*Mythimna separata*	Hadeninae	15,332	Li et al. (2015)

## Results

### Characteristics of mitochondrial genome

**Figure 2.**
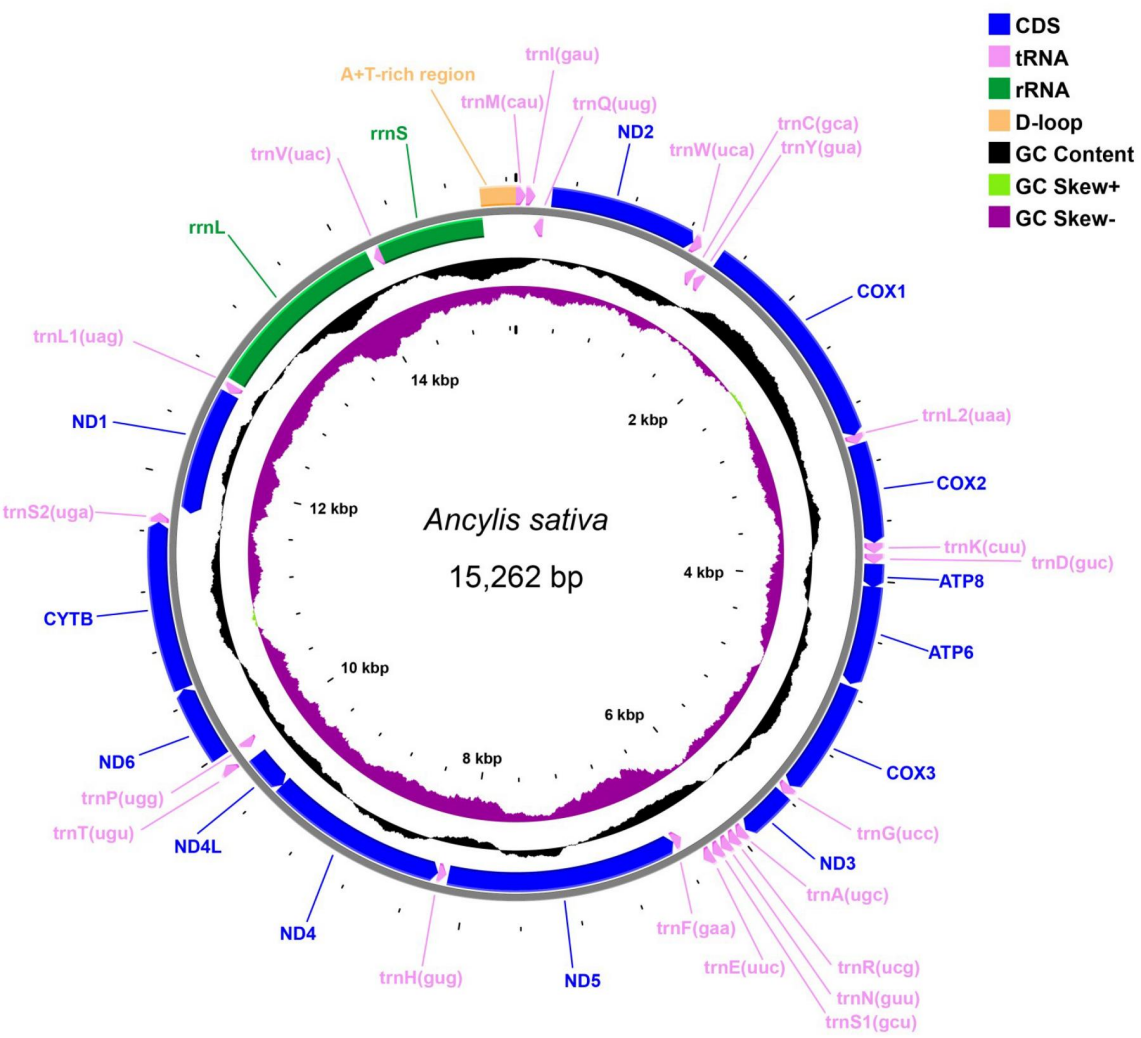
The mitochondrial genome map of *Ancylis sativa* with 13 PCGs, 22 tRNAs, two rRNAs, and one AT-rich region.

The mitogenome of *Ancylis sativa* is a double-stranded circular molecule with 15,262 bp in length (GenBank accession no. OQ865260), containing 37 genes (13 PCGs, 22 tRNAs, and two rRNAs) and one non-coding region (control region) (Figure 2). The average coverage of the mitogenome is 995.47× (Figure S1). The nucleotide composition is significantly biased (40.95% for A, 38.04% for T, 13.01% for C, and 8.00% for G). The percentage of A + T contents is 78.99%. AT-skew and GC-skew are 0.037 and −0.239, respectively. There are 19 intergenic spacer regions ranging in size from 1 to 57 bp (277 bp in total) and nine overlapping regions (31 bp in total) throughout the whole genome.

Among the 13 PCGs, *COX1* uniquely initiated with the CGA codon, while all other PCGs use standard ATN initiation codons: *ND6* (ATA), *ND2*, *ND3*, *ND5* (ATT), *ATP8* (ATC), and *COX2*, *ATP6*, *COX3*, *ND4*, *ND4L*, *CYTB*, *ND1* (ATG). All PCGs terminate with TAN stop codons: 10 PCGs (*ND2*, *COX1*, *ATP8*, *ATP6*, *COX3*, *ND3*, *ND4L*, *ND6*, *CYTB*, and *ND1*) with TAA, and three PCGs (*COX2*, *ND5*, and *ND4*) with an incomplete stop codon T. The 22 tRNAs range from 63 to 71 bp, all forming typical cloverleaf structures except *trnS1*, which lacks a DHU arm. Both rRNAs (*rrnL* and *rrnS*) are encoded on the N-strand. *rrnL* (*16S rRNA*) is 1356 bp in length with A + T contents of 83.55%, and *rrnS* (*12S rRNA*) is 781 bp in length with A + T contents of 84.89%. An AT-rich region (242 bp, 94.63% A + T) resides between *trnI* and *rrnS* (Figure 2).

### Phylogenetic analysis

**Figure 3.**
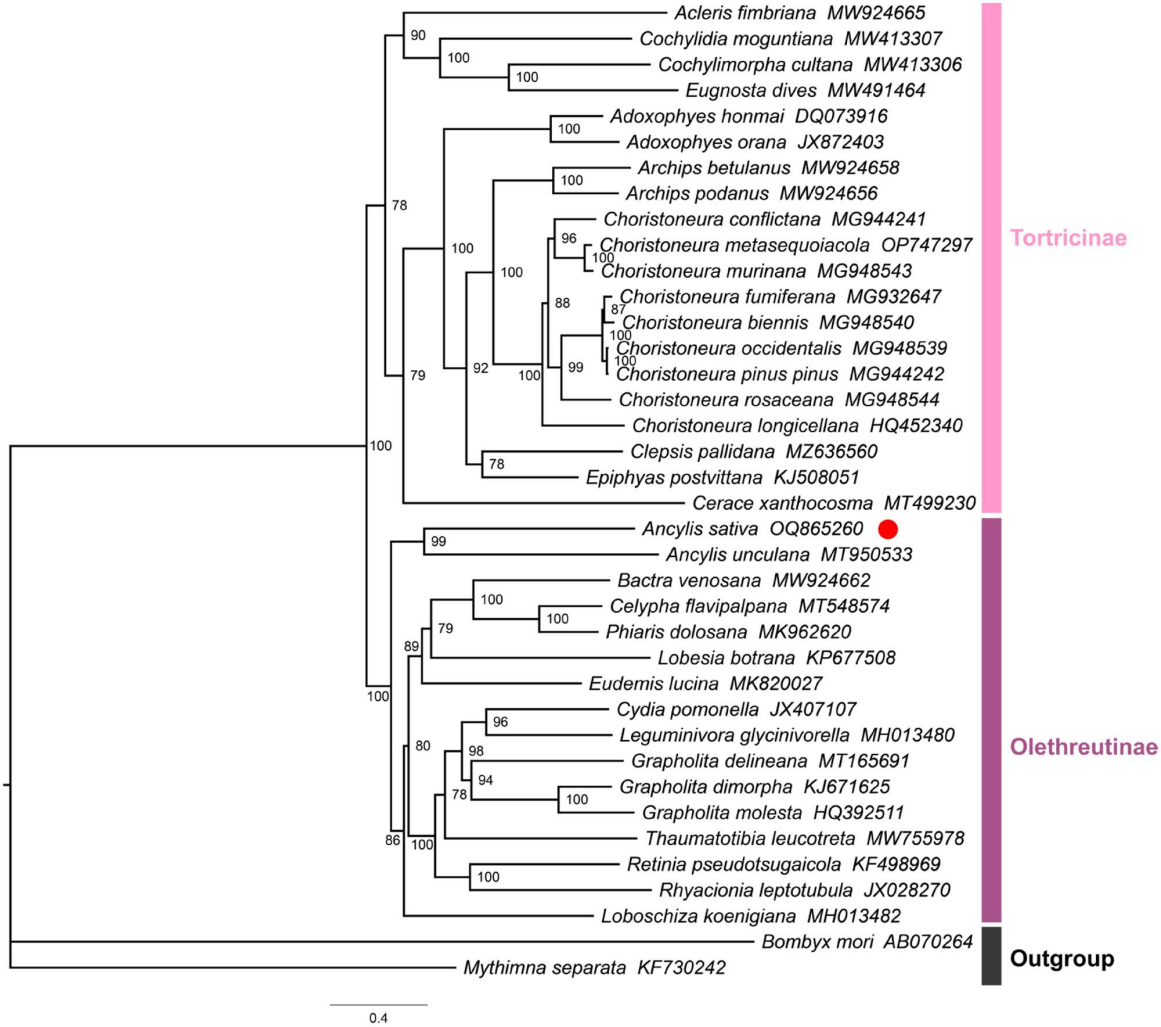
The maximum-likelihood tree of *Ancylis sativa* with 35 species in the family Tortricidae based on the concatenated nucleotide sequences of 13 PCGs. Bootstrap values were shown for each node.

Phylogenetic analysis of *Ancylis sativa*, along with 35 species in the family Tortricidae, was performed using the maximum-likelihood method; with *Bombyx mori* (Bombycidae: Bombycinae) and *Mythimna separata* (Hadenidae: Hadeninae) being used as outgroup species. The phylogenetic tree resolved *Ancylis sativa* and *Ancylis unculana* as sister taxa in the subfamily Olethreutinae, with high bootstrap values (BS = 99), indicating closer relationship than to other species (Figure 3).

## Discussion and conclusions

In this study, we assembled and described the characteristics of the first mitochondrial genome of *Ancylis sativa*. The gene arrangement of the mitogenome of *Ancylis sativa* is identical to that of most lepidopteran mitochondrial genomes in the ‘*trnM*-*trnI*-*trnQ*’ cluster (Cao et al. 2012). The phylogenetic analysis divided 36 species into two clusters, corresponding to subfamilies Tortricinae and Olethreutinae. Additionally, according to the phylogenetic results, *Ancylis sativa* showed a sister relationship with *Ancylis unculana* in the subfamily Olethreutinae. The phylogeny inferred that they may share a common ancestor, which deserves further study. Our research delivers essential molecular information that can enhance the evolutionary and phylogeographic study of *Ancylis sativa*, offering valuable perspectives for subsequent studies.

## Supplementary Material

supplementary_materials.docx

## Data Availability

The genome sequence data that support the findings of this study are openly available in GenBank of NCBI at https://www.ncbi.nlm.nih.gov/ under the accession no. OQ865260. The associated BioProject, Bio-Sample, and SRA numbers are PRJNA1207775, SAMN46143736, and SRR31924333, respectively.
