# Supplementary material for: The complete mitochondrial genome of jujube leaf roller, Ancylis sativa (Lepidoptera: Tortricidae) with phylogenetic implications
Source: Mitochondrial DNA B Resour. 2025 Oct 28;10(11):1062–7. doi: 10.1080/23802359.2025.2576516 (PMC12570247; doi:10.1080/23802359.2025.2576516)
Supplement: supplementary_materials.docx [file TMDN_A_2576516_SM8521.docx]

**The complete mitochondrial genome of** **jujube leaf roller, *Ancylis sativa* (Lepidoptera: Tortricidae) with phylogenetic implications**

Dongping Cao^a^, Bo Hong^b^, Li Dai^c^, Yingyan Zhai^b^, Tianqi Tian^b^ and Feng Zhang^b*^

*^a^**Forestry Workstation of Yulin City, Yulin, China; ^b^**Bio-Agriculture Institute of Shaanxi, Shaanxi Academy of Sciences, Xi'an, China; ^c^**Forestry & Grassland Pest Control and Fire Prevention Center of Dingbian County, Yulin, China*


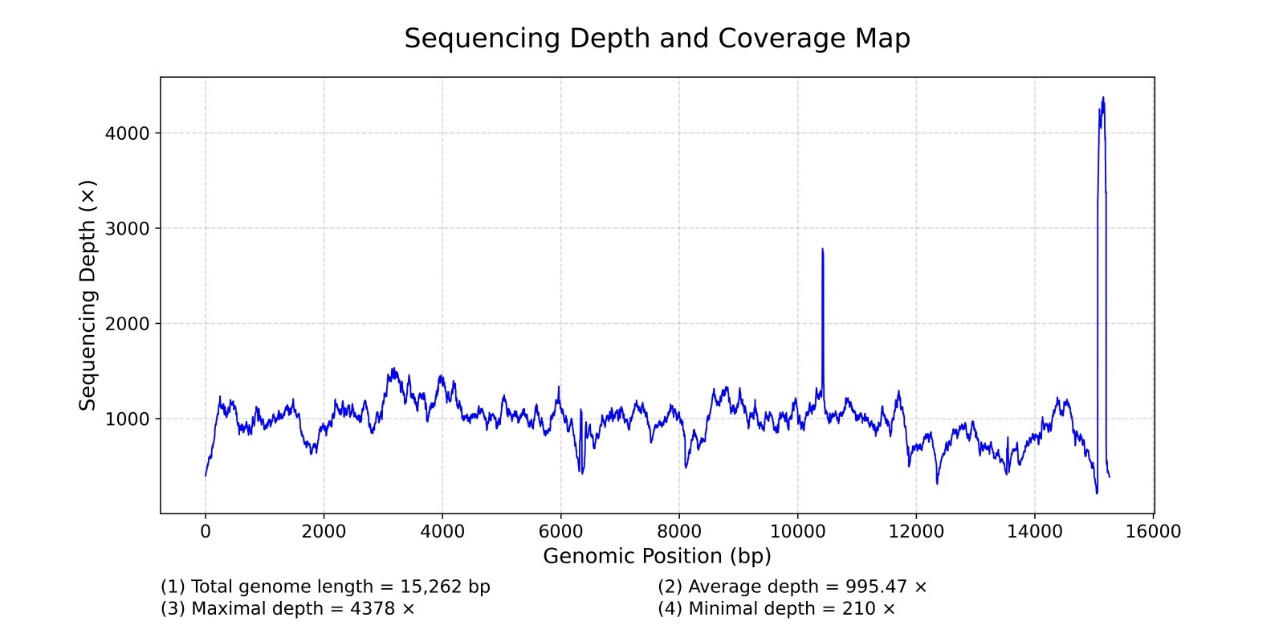


Figure S1. Clean reads coverage depth map of *Ancylis sativa*.
